# Real-world performance of the SSI Mantra™ robotic system: a multi-centric multi-specialty study evaluating its safety and surgical applications

**DOI:** 10.1007/s11701-025-03118-2

**Published:** 2026-01-03

**Authors:** Somashekhar SP, Medha Sugara, Kushal Agrawal, Sudhir Kumar Rawal, Amitabh Singh, Magan Mehrotra, Raj Gajbhiye, Chandramohan Vaddi, Srikarthik Voleti, Leena Mehrotra, Ganesh Gorthi, Manjiri Somashekhar, Nitin Kumar Rajput

**Affiliations:** 1 Department of Surgical Oncology, Aster International Institute of Oncology, Aster Hospital, Aster CMI Hospital, No. 43/2, New Airport Road, NH44, Sahakar Nagar, Hebbal, Bangalore, 560092 India; 2 Department of Genito-Uro-Oncology, Rajiv Gandhi Cancer Institute, New Delhi, India; 3 Department of Minimal Access and Bariatric Surgery, Apex Hospital, Moradabad, India; 4 Department of General Surgery, Government Medical College, Nagpur, India; 5 Department of Urology, Preeti Urology and Kidney Hospital, Hyderabad, India; 6 Department of Gynecology, Apex Hospital, Moradabad, India; 7 Department of Surgical Gastroenterology, Continental Hospital, Hyderabad, India; 8 Department of Pediatric Surgery, Aster International Institute of Oncology, Bangalore, India; 9 Department of Cardiothoracic Surgery, Narayana Institute of Cardiac Sciences, Narayana Health, Bengaluru, India

**Keywords:** Robotic-assisted surgery, SSI Mantra™, Console time, Conversion, Length of hospital stay, Surgeon experience, Learning curve

## Abstract

Robotic-assisted surgery (RAS) in India remains limited by cost and accessibility. The SSI Mantra™ is an indigenous system that was developed to address these barriers. This retrospective cohort study (IDEAL Stage 2b) analyzed 3,694 consecutive RAS cases across 78 centers in India (Feb 2023–Apr 2025) to assess the feasibility, safety, and learning curves of the system. Patient demographics, intraoperative metrics, and outcomes were extracted from prospectively maintained databases. Surgeons were grouped by experience quartiles (Q1–Q4) to analyze console time, conversions, and length of hospital stay (LOS) using descriptive and adjusted regression models. Learning curves were evaluated using continuous cumulative sum (CUSUM) analysis. Mean age was 51 years ± 15.8, with 57.2% being male. Mean blood loss was 75.4 ml ± 52.6; intraoperative adverse events (AEs) occurred in 0.6% of cases. Median LOS was 3 days (IQR: 2–4). Overall conversion rate was 4.1% (95% CI: 3.6–5.0%), varying by the procedure type and surgeon’s experience. More experienced surgeons (Q4) had shorter adjusted console times (154 vs. 169.6 min, *p* < 0.001) and lower conversion rates. Longer console time was linked to lower conversion odds. LOS increased with conversions but was more stable for experienced surgeons. CUSUM charts showed clear learning curve differences. No device-related AEs were reported. These results suggest that SSI Mantra™ is feasible, with minimal intraoperative AEs for diverse and complex procedures, with consistent performance across centers and specialties. Its lower capital cost may expand RAS access in India. Surgeon experiences improved efficiency and outcomes, supporting structured training. Long-term outcomes and comparative effectiveness warrant further study.

**Supplementary Information:**

The online version contains supplementary material available at 10.1007/s11701-025-03118-2.

## Introduction

Robot-assisted surgery (RAS) is an advanced form of minimally invasive surgery that integrates robotics, engineering, and medical expertise. This approach utilizes specialized robotic systems to enhance surgeon accuracy, particularly in intricate procedures and confined anatomical regions. By eliminating natural hand tremors and increasing dexterity, RAS helps minimize unintended movements. This increased precision often results in reduced surgical complications, including lower risk of surgical site infections, decreased pain and bleeding, shorter hospital stays, faster patient recovery, and smaller, less visible scarring [1]. Consequently, the robotic surgery market is expanding rapidly, with ~ 1.24 million procedures conducted across various surgical specialties in 2020 alone [2]. More than two decades since the introduction of the da Vinci system (Intuitive Surgical Inc, California, USA), robotic surgery has evolved considerably. Several alternative systems have since emerged, including the Hinotori™ surgical robot system (Medicaroid Inc., Japan), Hugo™ RAS (Medtronic, USA), Micro Hand S™ (Wego, China), Versius™ (CMR Surgical, UK), Senhance™ (formerly ALF-X) (Asensus Surgical, USA), and Revo-i™ (Meerecompany, Inc., South Korea) [3]. These platforms address the diverse needs of various surgical procedures and incorporate advancements in technology alongside customization. They also address the demand for modular and compact systems suited to different surgical setups across developing regions, while reducing cost, improving accessibility, and optimizing resource utilization [3]. Additionally, the expiration of key patents held by early market leaders has opened the door for competition, spurring the development of platforms that offer tailored solutions across various specialties and healthcare settings, especially in cost-sensitive markets [4].

Established and new surgical robotic systems have demonstrated consistent clinical effectiveness across a wide range of procedures, supporting their growing role in minimally invasive surgery. However, several limitations persist, particularly in low-resource settings. These challenges include high costs for acquisition, maintenance, disposable instruments, limited portability, dependency on proprietary consumables, and the absence of haptic feedback [5]. Furthermore, the requirement for extensive surgeon and team training, as well as limited interoperability with existing hospital infrastructure, hinders broader adoption. In many regions, infrastructure constraints and lack of technical support remain major barriers to implementation. Recent literature emphasizes the need for context-specific innovations and long-term multicenter studies to assess usability, cost-effectiveness, and sustainability in diverse healthcare environments [5]. Addressing these limitations is critical for expanding the benefits of robotic surgery beyond high-income regions.

To address the existing evidence gap, this retrospective observational cohort study evaluates the real-world performance, safety, and feasibility of the SSI Mantra™ (Sudhir Srivastava Innovations Pvt. Ltd, Haryana, India) conducted across diverse surgical specialties and healthcare settings in India. It is a modular robotic surgical platform comprising a surgeon console, a vision system, and multiple independent robotic arm carts, which support up to five arms and a range of commonly used robotic instruments across various surgical specialties. It is an advanced robotic surgical system developed in India to deliver cost-effective and accessible robotic surgeries globally, thus enhancing surgical precision, efficiency, and patient outcomes [6]. These system characteristics are described here to provide technical context; however, ergonomic performance and visualization advantages were not formally evaluated as outcomes in the present study. With 80 installations to date, over 3,500 surgeries performed, and more than 700 robotic surgeons trained on the SSI Mantra™, the system is gaining widespread adoption [7, 8]. Its adoption appears to be associated with its reported cost profile and applicability across a wide range of procedures [6]. The IDEAL framework guides the evaluation of surgical innovations through five structured phases: Idea, Development, Exploration, Assessment, and Long-term study [9]. This study aligns with the exploration Stage 2b, in which multicenter observational studies are encouraged to understand the safety, variability in practice, early outcomes, and learning effects.

## Materials and methods

### Study design and setting

This retrospective cohort study analyzed prospectively maintained data for 3,694 consecutive RAS performed using the SSI Mantra™ surgical robotic system across 78 centers in India. Participating centers spanned secondary and tertiary care institutions, both teaching and non-teaching, public and private hospitals. These centers were geographically distributed across public metro, as well as tier II (population of 50,000–99,999) and tier III (population of 20,000–49,999) cities, representing the East, West, North, and South regions of India (Supplementary Table S1).

### Participants

The study included all patients who underwent RAS with the SSI Mantra™ system between February 2023 and April 2025. Cases were excluded if they involved hybrid procedures with only brief robotic assistance or had incomplete intraoperative or postoperative data. A total of 302 surgeons participated, with individual case volumes ranging from 1 to 130 during the study period.

### Data collection

Data were extracted from institutional databases and medical records, and were then digitized using Microsoft^®^ Excel from Microsoft 365 (version 2206 - Build 15330.20306, Microsoft Corporation, Redmond, Washington, USA, 2022). Data were stored securely without personal identifiers. Variables included patient demographics, surgical specialty, procedure type, console time (as a proxy for operative duration), estimated blood loss (EBL), intraoperative AEs, conversions to open surgery, and length of hospital stay (LOS). All centers had at least one trained surgeon, one scrub nurse, and a technician using the SSI Mantra™ system.

### Ethics approval

The study was performed in accordance with the ethical standards as laid down in the 1964 Declaration of Helsinki and its later amendments or comparable ethical standards. The study was approved by the institutional ethics committee (reference number Aster/IEC/IIS/005/2024-25).

### Statistical analysis

Descriptive statistics summarized patient, procedural, and surgeon characteristics. Continuous variables were reported as mean ± standard deviation (SD) or median with interquartile range (IQR), depending on data distribution; categorical variables were summarized as frequencies and percentages. No imputation was performed for missing data.

For analytical purposes, procedures were grouped into surgical specialties based on institutional classification. General surgery included procedures such as cholecystectomy, hernia repair, and appendectomy. Gastrointestinal surgery comprised upper gastrointestinal and hepatopancreatobiliary procedures, including gastrectomy, fundoplication, pancreatic resections, and biliary reconstructions. Colorectal surgery included procedures involving the colon and rectum, such as colectomy, low anterior resection, and abdominoperineal resection. These categories were mutually exclusive for analysis, although clinical overlap exists between specialties.

Surgeons were stratified into quartiles of experience based on their total robotic case volume during the study period. Q1, Q2–Q3, and Q4 constituted surgeons with the lowest 25%, middle 50%, and highest 25% of the total case volume, respectively. For analytical purposes, surgeons in Q1–Q3 were grouped as “less experienced”, while those in Q4 were classified as “more experienced”. This approach ensured stable estimates while supporting a clear contrast in cumulative experience. Comparisons of the mean console time between groups were performed using linear regression, adjusted for procedure type. Assumptions of linearity, homoscedasticity, and residual normality were verified using diagnostic plots.

Conversion rates were calculated overall, by surgical specialty, and by procedure type. The association between console time and conversion risk was assessed using multivariable linear regression, adjusting for surgeon experience and procedure specialty. Model robustness and calibration were confirmed prior to interpretation.

The LOS was analyzed as an indicator of postoperative recovery. Due to its right-skewed distribution, it was summarized using the median (IQR) and compared between converted and non-converted cases using negative binomial regression, adjusting for procedure, console time, and surgeon experience. An interaction term was assessed to determine whether the effect of conversion on LOS differed by surgeon experience.

The top five specialties with the highest caseloads were identified, and the cumulative sum (CUSUM) analysis was performed to evaluate temporal trends in console time as a surrogate of operative efficiency. For each procedure-specific analysis, cases were ordered chronologically by the date of surgery. Console time was expressed in minutes, and a reference value was defined as the overall mean console time for that procedure. The CUSUM statistic for each case i was calculated as: CUSUM_i_ = Σ (X_i_ − µ), where X_i_ is the observed console time for case i and µ is the mean console time for the corresponding procedure. Separate CUSUM curves were generated for surgeons classified as novice (Q1–Q3) and experienced (Q4), based on the procedure-specific case-volume quartiles.

CUSUM plots were used descriptively to assess learning trends and changes in the operative efficiency over time. Formal decision limits or stopping boundaries were not applied, as the objective was exploratory visualization of performance trajectories rather than hypothesis testing or real-time monitoring.

Scatter plots and box plots were generated to visually represent distributions and associations.

All analyses were conducted using RStudio version 2024.12.1 + 563 (Posit, USA). Statistical significance was set at *p* < 0.05.

## Results

### Overall cohort characteristics and case mix across specialties

A total of 3,694 patients underwent robotic procedures during the study period. The mean patient age was 51.0 years **±** 15.8, with 57.2% of patients being male and 42.8% female. The mean EBL across all robotic procedures was 75.4 ml **±** 52.6, indicating minimal intraoperative bleeding typical of minimally invasive surgery. The median LOS was 3 days (IQR: 2–4). The overall conversion rate from robotic to open surgery was 4.1%. Intraoperative AEs were rare, occurring in 17 (0.6%) cases across all procedures. The mean number of robotic instruments used per procedure was 3.4 **±** 0.7, with a median of 3(3–4). Most procedures were performed using four robotic arms (74.2%), followed by three-arm configurations (25.7%); five-arm configurations were rarely used (0.1%). The use of assistant ports was variable, left to the surgeon’s discretion, and was not systematically recorded for all cases. As such, assistant port utilization was not included in the formal analysis.

**Table 1:** Descriptive statistics for all the patients

Characteristics	Value
Mean age, years (SD)	51.0 (15.8)
Sex, n (%)	Male: 2113 (57.2%) Female: 1581 (42.8%)
Specialty, n (%)	General Surgery: 1499 (40.6%) Urology: 1046 (28.3%) Gynecology: 548 (14.9%) Gastrointestinal Surgery: 115 (3.1%) Cardiac: 195 (5.3%) Colorectal: 201 (5.4%) Thoracic: 37 (1.0%) Head & Neck: 48 (1.3%) Breast & Plastic Surgery: 4 (0.1%) Other: 1 (0%)
Mean EBL, ml (SD)	75.4 (± 52.6)
LOS, days	Median (IQR): 3 (2–4)
Conversion to open, n (%)	152 (4.1%)
Intra-operative adverse events, n (%)	17 (0.6%)
Number of robotic arms used, n (%)	3-arm: 950 (25.7%) 4-arm: 2741 (74.2%) 5-arm: 3 (0.1%)
Number of robotic instruments used	Mean (SD): 3.4 (0.7) Median (IQR): 3 (3–4)

No imputation was performed for missing data. *EBL* estimated blood loss; *IQR* Interquartile range; *LOS* length of hospital stay; *SD* standard deviation

General Surgery represented the largest share (40.6%), followed by urology (28.3%) and gynecology (14.9%) (Table 1). Cardiac procedures accounted for 6%, colorectal procedures for 5.4%, and gastrointestinal procedures for 3.1%. Thoracic, head & neck, and breast & plastic surgeries accounted for less than 2% of the total caseload combined. This distribution highlights the adoption of robotic surgery across multiple specialties, with general surgery and urology contributing the highest procedural volumes.

### Experienced surgeons have shorter console times

A total of 3,694 robotic procedures were analyzed, focusing on the console time and conversion outcomes across surgeon experience levels. Surgeons were stratified into experience groups based on the total case volume: Q1–Q3 were defined as the less experienced group, representing surgeons with low to moderate cumulative robotic exposure. Q4 was defined as the more experienced group, representing surgeons with the highest cumulative procedure volumes. This ensured a sufficient sample size for stable estimates while retaining a clear experience gradient. Particularly, since the number of individual surgeons in Q1–Q3 was relatively small. Overall, Q4 surgeons demonstrated shorter operative console times than Q1–Q3.

**Fig. 1:**
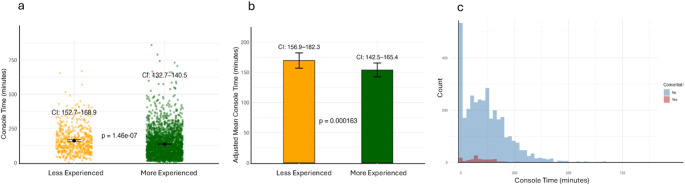
Console time analysis by surgeon experience. **a** raw jittered plot showing console time for individual procedures, stratified by surgeon experience quartiles (Q1–Q3 as less experienced vs. Q4 as more experienced), **b** procedure-adjusted mean console time (with 95% confidence intervals) comparing less experienced and more experienced surgeons, estimated using linear regression, **c** histogram of console time distribution for all cases, overlaid with the number of conversions per console time bin (converted cases shown in red)

The distribution of console time across individual procedures, shown as raw jittered points (Fig. 1a), indicates a clear separation by experience quartiles. The unadjusted mean console time in Q1–Q3 was 160.8 min (95% CI: [152.7–168.9]), compared to that of 136.6 min (95% CI: [132.7–140.5]) in Q4 (*p* < 0.001). This indicates improved efficiency with accumulated experience.

To account for potential confounding by the procedure type, we adjusted console time estimates using linear regression (Fig. 1b). The visual inspection of residual plots indicated no substantial deviations from linearity or homoscedasticity (Supplementary Fig. 1). Residuals were approximately normally distributed. A few high-residual data points were identified but were not influential. The difference persisted, with an adjusted mean console time of 154 min (95% CI: [142.5–165.4]) for Q4 surgeons versus 169.6 min (95% CI: [156.9–182.3]) for Q1–Q3 surgeons (*p* < 0.001). The adjusted mean difference was − 15.6 min (95% CI: − 23.7 to − 7.5). This suggests that the effect of experience on efficiency remains consistent across surgical classes.

The histogram shows the distribution of console times for all cases, with the number of converted cases overlaid for each console time bin (Fig. 1c). The converted cases (highlighted in red) clustered predominantly within the lower console time bins, indicating that conversions tended to occur early in the surgical procedure. This suggests that conversions were not driven by prolonged console time but were likely due to intraoperative factors identified at the start of the operation, demonstrating timely surgical decision-making.

### Conversions cluster early and vary by experience, procedure, and console time

**Fig. 2:**
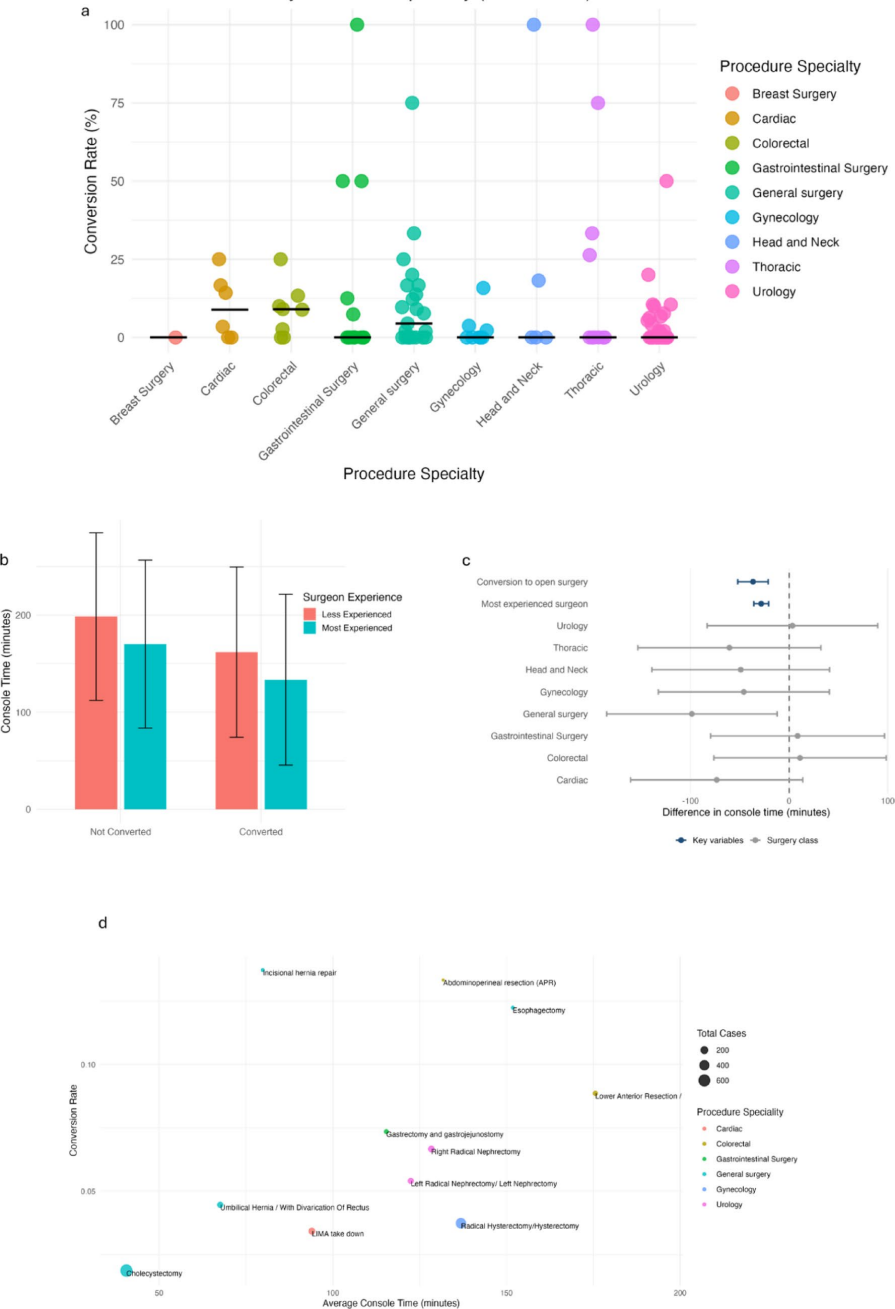
Console time and conversion analysis. **a** Conversion rate (%) by surgical class, **b** bar plot showing the adjusted mean console time (min) with 95% confidence intervals, stratified by conversion to open surgery and surgeon experience group. Estimates are derived from a multivariable linear regression model with console time modeled as the outcome and adjustment for procedure specialty and surgeon experience, **c** forest plot showing adjusted regression coefficients (β) from a multivariable linear regression model with console time (min) as the outcome. Points represent adjusted mean differences in console time, and horizontal lines indicate 95% confidence intervals. Negative values indicate shorter console time relative to the reference category. The model was adjusted for procedure specialty and surgeon experience, **d** scatter plot showing the descriptive relationship between average console time and conversion rate across procedures

All procedures were completed with definitive surgical management; however, 4.1% (152 out of 3,694) (95% CI: [3.6%–5.1%]) required conversion to an open approach to safely complete the operation (Table 1). We next plotted the distribution of conversion rates by individual procedures across all major surgical specialties (Fig. 2a). Gynecology, urology, and thoracic surgeries showed moderate variability, while cardiac surgery demonstrated relatively low but consistent conversion rates overall. General, colorectal, and gastrointestinal surgeries exhibited the widest spread of conversion rates, reflecting greater procedural complexity and intraoperative challenges. In contrast, breast surgery and head & neck surgery consistently showed low conversion rates, with minimal variation across procedures. The pattern suggests that intraoperative factors driving conversion are strongly procedure-specific, while specialties with broader and more complex case mixes tend to show greater variation in conversion risk. In surgical procedures with ≥ 20 cases and ≥ 5 conversions, the highest conversion rates were observed in abdominoperineal resection and incisional hernia repair, both at 17.7% (95% CI: 8.4–33.5%), followed by esophagectomy (13.6%, 95% CI: 6.4–26.7%) and gastrectomy with gastrojejunostomy (9.8%, 95% CI: 4.3–21.0%) (Supplementary Figure S2). Procedures such as lower anterior resection (LAR), ultra LAR, and right radical nephrectomy also showed moderate conversion rates of 9.8% and 7.4%, respectively. In contrast, cholecystectomy and left internal mammary artery (LIMA) take down had the lowest conversion rates at 2.2% (95% CI: 1.2–3.8%) and 3.6% (95% CI: 1.6–8.1%), respectively. In multivariable linear regression analysis with console time modeled as the outcome, conversion to open surgery was associated with shorter operative duration (Fig. 2b and Supplementary Table S3). After adjustment for procedure specialty and surgeon experience, converted cases had a mean console time that was 36.7 min shorter than cases completed robotically (β = −36.7 min; 95% CI: −52.1 to − 21.3; *p* < 0.001). Surgeon experience was also significantly associated with operative duration, with procedures performed by the more experienced surgeons demonstrating a mean console time 28.4 min shorter than those performed by less experienced surgeons (β = −28.4 min; 95% CI: −35.8 to − 20.9; *p* < 0.001). Sensitivity analyses using log-transformed console time yielded consistent results, with conversion associated with an approximately 21% reduction in console time and surgeon experience with an approximately 30% reduction (Fig. 2c and Supplementary Table S4). Figure 2d displays the average console time versus conversion rate by procedure, demonstrating variability in conversion risk across surgical domains. While not adjusted for confounders, the plot highlights the interaction between procedural complexity and operative outcomes.

Overall, more experienced surgeons demonstrated shorter console times and lower conversion rates across procedures. Together, these findings indicate that conversion to open surgery typically occurs earlier in the operative course, consistent with timely intraoperative decision-making rather than prolonged robotic attempts. However, as console time reflects intraoperative processes, the influence of case complexity and unmeasured patient-level factors cannot be excluded.

### CUSUM charts show steeper learning curves for less experienced surgeons

**Fig. 3:**
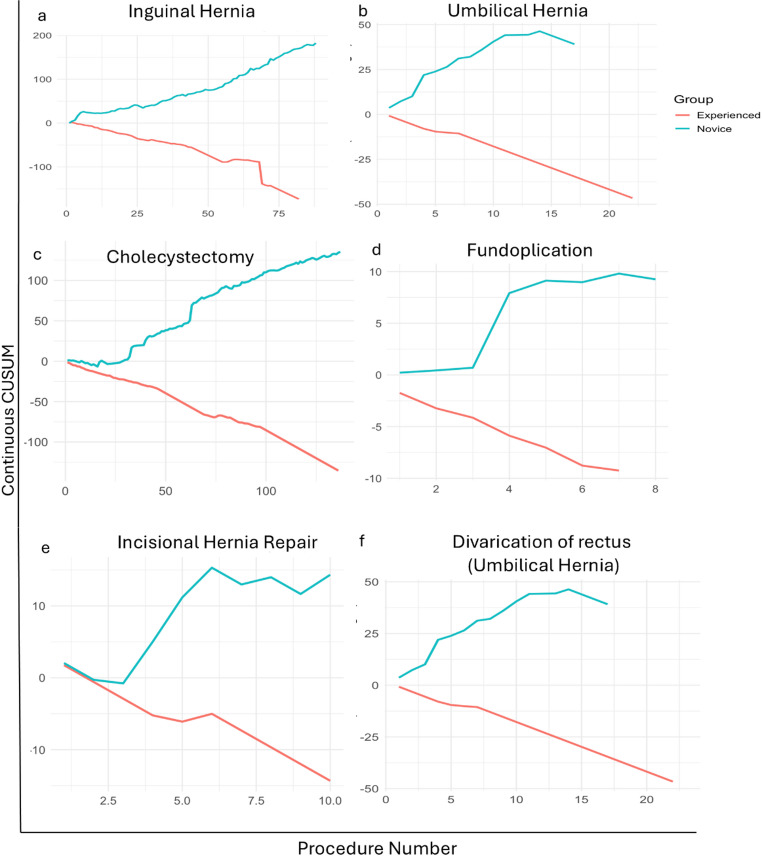
Continuous CUSUM plots of console time for key general surgery procedures comparing the least experienced surgeons (Q1) and the more experienced surgeons (Q4). **a** Inguinal hernia, **b** Umbilical hernia, **c** Cholecystectomy, **d** Fundoplication, **e** Incisional hernia repair, **f** Divarication of rectus (umbilical hernia)

We next performed continuous CUSUM analyses to illustrate surgeon learning curves for console time across six common general surgery procedures: inguinal hernia, umbilical hernia, cholecystectomy, fundoplication, incisional hernia repair, and divarication of rectus (Fig. 3).

To sharpen the contrast in learning patterns, surgeons were stratified into Q1 and Q4 based on the cumulative robotic case volume. Q1 represents surgeons with the least overall experience, capturing the earliest phase of the learning curve. Q4 represents surgeons with the highest cumulative procedure volume, serving as a benchmark for full proficiency. This approach differs intentionally from the preceding section, where Q1–Q3 was combined to provide stable mean estimates and sample size for mean estimate comparisons. For the CUSUM learning curve plots, however, comparing the extreme quartiles (Q1 vs. Q4) allows a clearer visualization of the efficiency gap and trajectory differences. All trajectories were plotted in the chronological order by the procedure date to maintain temporal fidelity. Across procedures, the Q4 group consistently demonstrated stable, downward-sloping CUSUM trajectories, reflecting shorter console times and early proficiency with the robotic platform. In contrast, the Q1 group displayed wider fluctuations and prolonged upward trends, indicating ongoing adaptation to the robotic system and greater variability in operative performance.

**Fig. 4:**
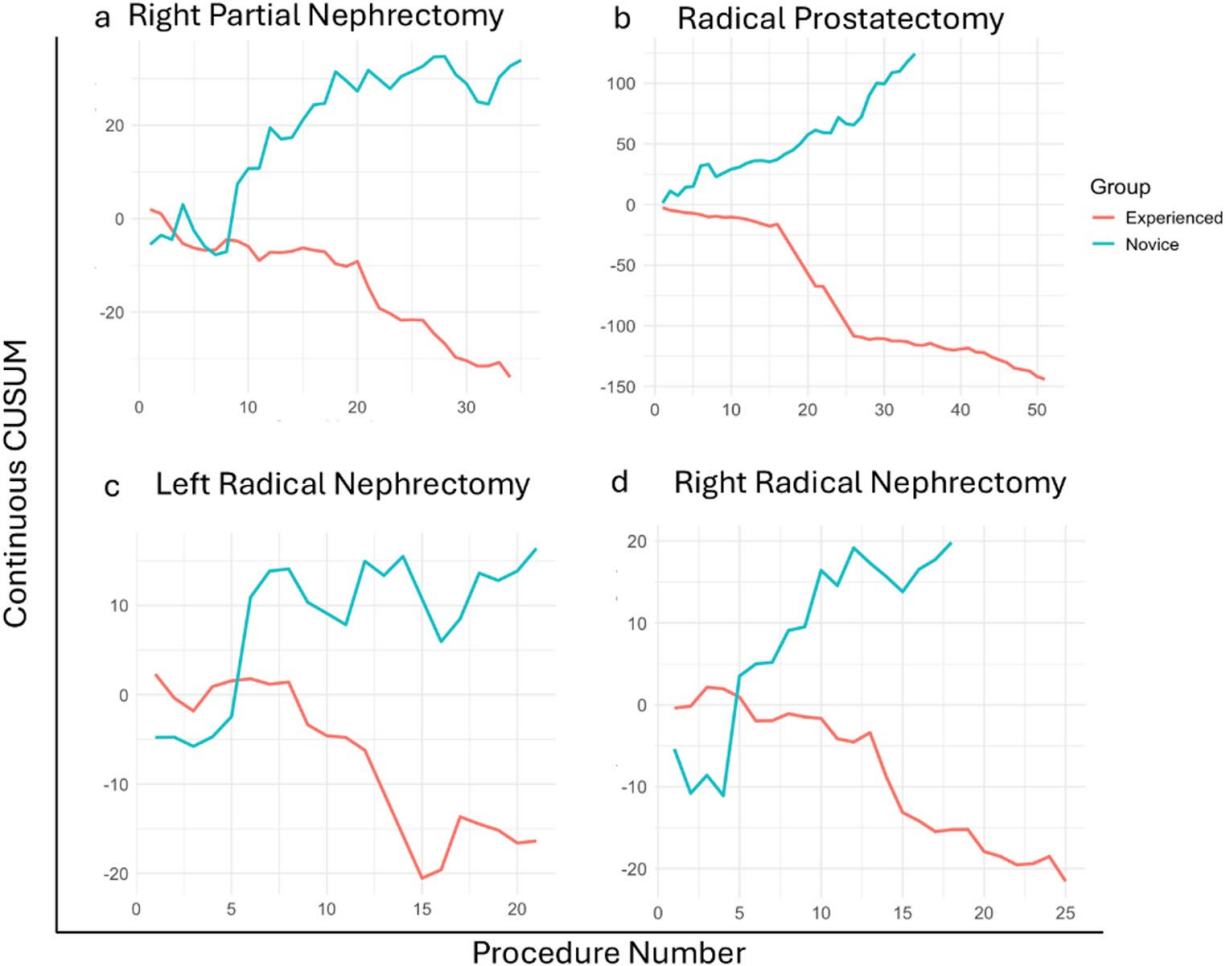
Continuous CUSUM plots of console time for key urology procedures comparing the less experienced surgeons (Q1) and the more experienced surgeons (Q4). **a** Right partial nephrectomy, **b** radical prostatectomy, **c** left radical nephrectomy, **d** right radical nephrectomy

We conducted a similar continuous CUSUM analysis to assess console time learning curves across urology procedures, specifically right partial nephrectomy, radical prostatectomy, left radical nephrectomy, and right radical nephrectomy (Fig. 4). Consistent with the general surgery findings, the Q4 surgeons maintained consistently stable, downward-sloping trends, indicating reliable procedural efficiency and mastery across these complex cases. Conversely, Q1 surgeons exhibited more variable and prolonged upward trends, indicating an extended learning phase with greater fluctuations in console time.

Additional continuous CUSUM charts for lower-volume procedures, including internal mammary artery (IMA) harvest (cardiac), lower anterior resection (colorectal), hysterectomy (gynecology), and gastrectomy/gastrojejunostomy (gastrointestinal), show similar trends for experienced vs. novice surgeons (Supplementary Figure S3).

These findings demonstrate a consistent trend of improved operative efficiency and stabilization of console time with increasing surgeon experience across multiple surgery classes, including general surgery, urology, cardiology, colorectal surgery, gynecology, and gastrointestinal surgery.

### Impact of conversion on LOS varies with surgeon experience

**Fig. 5:**
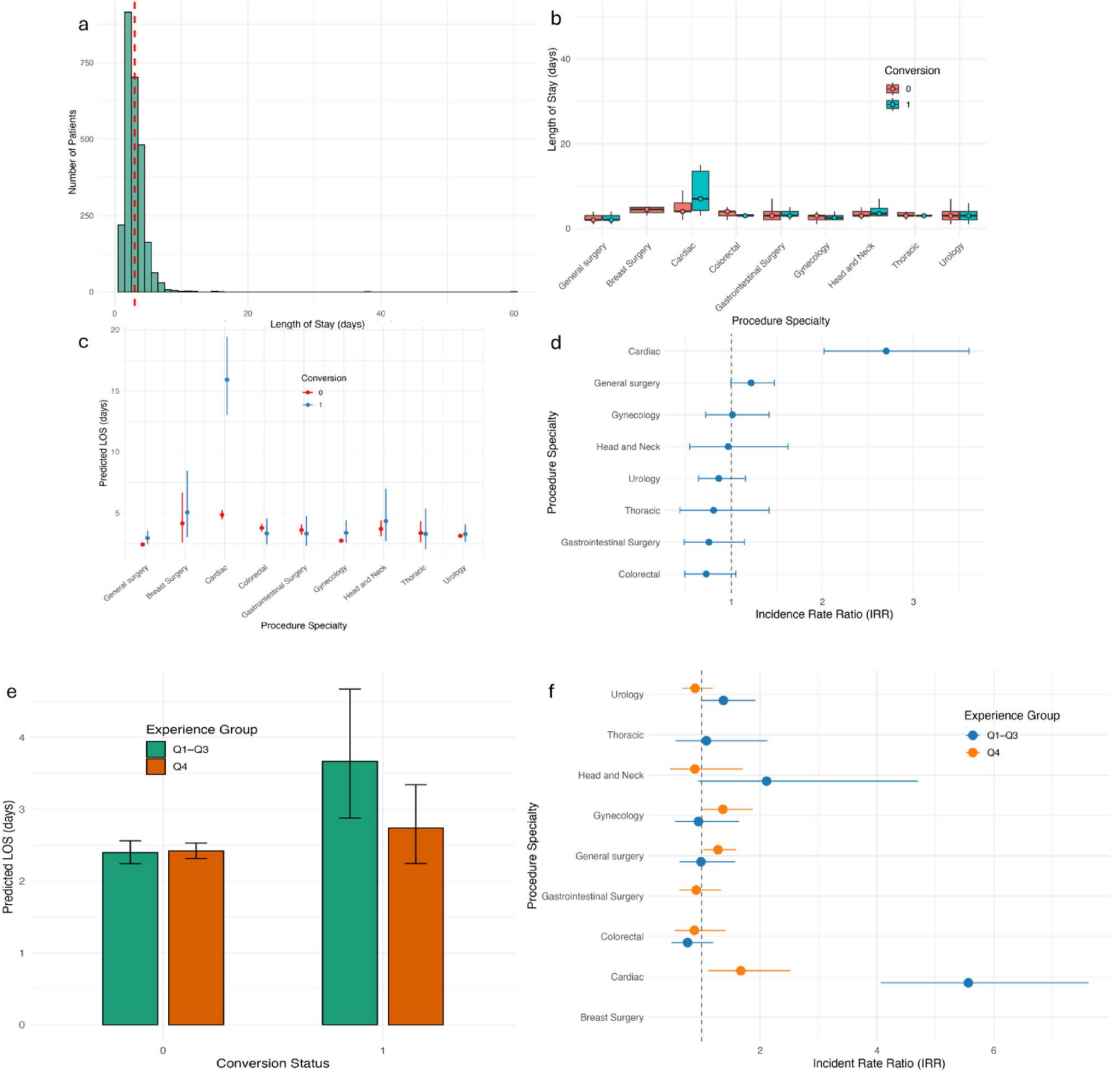
Length of hospital stay (LOS) comparisons by conversion status and other covariates. **a** Distribution of observed LOS (in days) across all included surgical classes, **b** Boxplots showing LOS distributions across surgical specialties, stratified by conversion. This represents raw, unadjusted comparisons (outliers removed for better visualization), **c** Predicted mean LOS values from a negative binomial regression model adjusted for procedure specialty, conversion status, and console time. Bars represent predicted LOS with 95% CIs, **d** Adjusted IRRs with 95% confidence intervals from a negative binomial regression model assessing the association between conversion and LOS, stratified by surgical specialty. An interaction term was included between conversion status and procedure specialty. IRRs > 1 suggest higher LOS with conversion, **e** Model-predicted LOS adjusted for console time and procedure specialty, stratified by conversion status and surgeon experience group, **f** Adjusted IRRs with 95% CIs for conversion effect on LOS, stratified by surgeon experience and surgical specialty. LOS: length of hospital stay; CI: confidence interval; IRR: incident rate ratio; Q: quartile

The influence of conversion and surgeon experience on postoperative LOS was assessed using both unadjusted and model-adjusted visualizations, with procedure specialty incorporated throughout (Fig. 5). The overall distribution of LOS was right-skewed, with most patients staying around 2–4 days and a long tail extending to 60 days (Fig. 5a). This was further summarized using a box plot with a median LOS of 3 days (IQR: 2–4) (Fig. 5b). Descriptive analysis stratified by surgeon experience (Supplementary Table S2) revealed that among Q1–Q3 surgeons, the converted cases had a higher mean LOS (5.0 vs. 3.0 days), greater variability (SD: 9.5 vs. 2.0), and a wide range extending up to 60 days. In contrast, the difference was modest in Q4 (3.3 vs. 3.0 days), and the LOS distribution was more consistent (1–15 days). These findings suggest that while conversion to open surgery generally increases LOS, surgeon experience appears to mitigate both the magnitude and variability of this impact.

To further evaluate the effect of conversion across specialties, we plotted model-predicted LOS estimates across surgical classes stratified by conversion status. Estimates were derived from a negative binomial regression adjusting for procedure specialty and console time (Fig. 5c). Converted cases had higher predicted LOS across nearly all specialties. The difference was most pronounced in cardiac surgery, while smaller differences were seen in gynecology and urology. These adjusted predictions support the general association between conversion and prolonged LOS, while also highlighting inter-specialty variability. The adjustment for surgeon experience ensures that these effects are not confounded by operator-level differences. Figure 5d presents a complementary forest plot showing the relative effect of conversion across specialties, expressed as incidence rate ratios (IRRs) from the same adjusted regression model. While the previous figure displays absolute predicted LOS, this plot emphasizes the relative impact of conversion within each specialty. General surgery was used as the reference group, with its IRR manually added for comparison. Conversion to open surgery was associated with a statistically significant increase in LOS only in cardiac surgery (IRR: 2.7; 95% CI: 2.0–3.6; *p* < 0.001), suggesting a 170% longer stay compared to non-converted cases. For other specialties such as colorectal (*p* = 0.096), gastrointestinal surgery (*p* = 0.198), and gynecology (*p* = 0.940), the IRRs were not statistically significant, with confidence intervals crossing 1. Although the direction of effect remained consistent (longer LOS with conversion), statistical significance varied, underscoring heterogeneity in the impact of conversion across surgical domains.

We next explored whether the impact of conversion on LOS varied by surgeon experience (Fig. 5e). Among less experienced surgeons, conversion was associated with a substantial increase in adjusted LOS. In contrast, among the more experienced surgeons (Q4), the predicted LOS remained comparatively stable between converted and non-converted cases. To further assess how surgeon experience modifies the impact of conversion across specialties, we fitted a second negative binomial regression model including an interaction term between conversion status and experience group (Fig. 5f). Among Q1–Q3 surgeons, conversion was associated with markedly increased LOS in certain specialties. For instance, cardiac surgery showed a nearly 5.6-fold increase in LOS (IRR: 5.6; 95% CI: 4.0–7.6), while head & neck surgery also exhibited a substantial effect (IRR: 2.1; 95% CI: 0.95–4.7), although not statistically significant. In contrast, other specialties, such as colorectal and gynecology had IRRs closer to 1, indicating smaller or null effects of conversion among less experienced surgeons. In the Q4 group, the relative effect of conversion was generally attenuated and more consistent across specialties. Notably, the IRR for cardiac surgery dropped to 1.67 (95% CI: 1.1–2.5), and estimates for general surgery (IRR: 1.3; 95% CI: 1.0–1.6) and gynecology (IRR: 1.4; 95% CI: 0.99–1.9) suggested more modest increases in LOS. Several specialties, such as colorectal, head & neck, and urology showed IRRs below or near 1, suggesting minimal additional LOS burden with conversion when performed by experienced surgeons. These results align with the descriptive findings and suggest that while conversion prolongs LOS, experienced surgeons may buffer its impact, potentially through more efficient intraoperative decision-making.

We next generated a radar plot (Supplementary Figure S4) to visually consolidate key metrics: console time, conversion rate, and LOS, across surgeon experience levels. In the raw values plot (Supplementary Figure S4a), Q4 surgeons had shorter console times, lower conversion rates, and reduced LOS compared to Q1–Q3. However, due to scale differences, console time visually dominated the plot. We next generated a normalized plot to achieve equal weightage across variables (Supplementary Figure S4b). The normalized plot visually reinforced prior analyses, which independently demonstrated that greater surgical experience is associated with improved operative efficiency and patient outcomes.

## Discussion

This large, multicenter, retrospective, observational cohort study provides a comprehensive real-world assessment of the safety, feasibility, and initial learning experience of the SSI Mantra™ surgical robotic system across diverse healthcare settings, including secondary and tertiary centers in metropolitan and tier II/III cities, and across public and private hospitals in India. The dataset includes 9 specialties and around 90 distinct procedures performed by roughly 300 surgeons at 78 centers on 3,694 patients. The broad distribution of specialties, including general surgery, gynecology, urology, surgical oncology, and cardiothoracic surgery, demonstrates the system’s adaptability and versatility [7, 8]. In Stage 2b, an exploration study within the IDEAL framework for surgical innovation, this early evaluation focused on safety, feasibility, and procedural learning curves across a wide spectrum of surgical applications [9].

Our categorization strategy, based on surgeon-specific case volume quartiles, aligns with published literature demonstrating the utility of stratifying operator experience using data-driven thresholds [10]. In our study, pooling surgeons into Q1–Q3 as less experienced and Q4 as more experienced for the console time, conversion, and LOS analyses ensured a stable estimate while maintaining a clear contrast in cumulative experience. For the learning curve analysis, the continuous CUSUM charts used the extremes (Q1 vs. Q4) to examine how performance evolved among the less and more experienced surgeons. This tiered approach allowed us to capture meaningful variation in performance that may be masked when using a simple chronological case-order split alone. Such surgeon volume-based stratification improves the interpretability of experience-related trends across real-world, multi-surgeon, multi-specialty robotic programs, particularly relevant for a new surgical robotic system like the SSI Mantra™, where feasibility and safety must be demonstrated across varying operator experience levels. The significant reduction in the mean console time for Q4 surgeons, both before and after adjustment for the procedure type, reinforces the well-documented impact of cumulative experience on operative efficiency [11]. This trend persisted across specialties, highlighting the importance of skill acquisition as robotic platforms are integrated into routine surgical practice.

Across the cohort, there were 17 documented intraoperative AEs. Notably, the overall conversion rate was 4.1%, comparable to rates reported for other early-phase robotic surgery programs [12–14]. The histogram analysis revealed that conversions were most frequent in procedures with shorter console durations, suggesting that intraoperative decisions to convert were made early, likely driven by anatomical or technical challenges rather than extended operative time. The multivariable linear regression further supports this interpretation. Conversions were also driven by surgical specialty, procedure type, and surgeon experience, rather than any single variable. The continuous CUSUM learning curve analysis strengthened these findings by showing clear contrasts in efficiency trajectories. Comparing the extreme quartiles (Q1 vs. Q4) allowed us to illustrate how less experienced surgeons displayed wider CUSUM fluctuations and prolonged upward trends, consistent with an extended learning phase. In contrast, Q4 surgeons maintained stable downward slopes, reflecting consistent proficiency and shorter console times. Thus, conversions are indeed influenced by a combination of surgery class, procedure, and surgeon adaptation, rather than any single variable alone [15, 16]. In this real-world cohort, the robotic system was feasible across the procedures studied.

LOS was used as a proxy for postoperative recovery and surgical safety. Most patients were discharged within 2–4 days; prolonged stays were primarily associated with conversion to open surgery. However, the magnitude of this increase varied by surgeon experience and procedure specialty. Among less experienced surgeons (Q1–Q3), converted cases had higher mean LOS and greater variability. In contrast, experienced surgeons (Q4) showed minimal LOS differences between converted and non-converted cases, suggesting that experience may buffer the impact of intraoperative challenges. Procedure specialty also influenced LOS outcomes. Converted cases in cardiac surgery had the highest predicted LOS, while other specialties, such as gynecology and colorectal surgery, showed smaller, often nonsignificant differences. Interaction models further revealed that the relative effect of conversion was most pronounced in Q1–Q3 surgeons, especially in cardiac and head & neck surgeries but attenuated among Q4 surgeons. These findings are within the expected range for robotic procedures [17]. These data reflect efficient perioperative care and suggest that the system facilitates early recovery and discharge, even in resource-constrained settings.

The mean EBL was low at 75.4 ml, consistent with the well-established advantage of robotic platforms in minimizing intraoperative bleeding through enhanced visualization and precise dissection. These findings align with reports from other robotic systems, which typically demonstrate mean EBL ranging from 50 to 150 ml for similar procedures [18, 19]. The comparable LOS, low EBL, and low intraoperative AEs observed in this study suggest that the SSI Mantra™ robotic system provides a level of perioperative safety.

Thus, our findings suggest that robot-assisted surgery using the SSI Mantra™ system is feasible and effective, with minimal intraoperative AEs, across a wide spectrum of surgical specialties and procedures. While general surgery and urology led in procedure volume, the system was also feasible in gynecology and upper gastrointestinal surgeries, where evidence suggests limited advantages over laparoscopy or open surgery [20]. A similar trend was documented in the Michigan Surgical Quality Collaborative registry, where the use of robotic surgery for all general surgical procedures increased from 1.8% in 2012 to 15.1% in 2018 [21]. Moreover, hospitals that initiated robotic surgery programs saw a broad and immediate increase in their use, often at the expense of traditional surgery. The system was successfully employed for complex surgeries, including Whipple’s pancreaticoduodenectomy and esophageal resection, with no device-related complications or surgery-related mortalities. In advancing minimally invasive surgical techniques, the robotic system was applied in confined anatomical spaces, such as thyroidectomy and nipple-sparing mastectomy. Unlike the abdominal approach, which relies on pneumoperitoneum to create a working space, these procedures require soft tissue expansion using low-pressure insufflation. This underscores the system’s versatility in unique operative settings.

A major requirement for any robotic surgery system is the ability to provide clear and effective visualization while maintaining the surgeon’s comfort and control, resulting in positive outcomes. The SSI Mantra™ system has an open console design that offers a wide-angle or bird’s-eye view without compromising magnification. The telescopic view in other systems requires the console surgeon to zoom out with multiple adjustments to achieve a similar wide-angle perspective, a difference that is most noticeable during procedures such as nephrectomy and esophageal mobilization. Although not directly measured in this study, the open console design helps improve ergonomics and facilitates better communication with the surgical team [22]. This configuration helps reduce the risks associated with vergence-accommodation and visual-vestibular mismatches, which are often problematic in immersive telescopic systems. Such mismatches can contribute to neck and trapezius strain for the surgeon from the need to lean into the console for extended periods. Future studies incorporating validated ergonomic and fatigue assessment tools are needed to objectively evaluate these potential advantages.

Since its first installation in 2006, India has had 100 robotic surgical systems, with over 50,000 procedures performed till 2021 [23]. Considering the population exceeding one billion, the overall adoption remains relatively limited. Several factors contribute to the limited adoption of robotic surgery across the country, including high implementation cost, shortage of trained personnel, and limited accessibility in disadvantaged communities [24, 25]. The initial investment for a robotic surgical platform can range from 2 to 3 million USD depending on the accessories (initial investment). Additionally, the recurring costs of instrumentation, disposables, ongoing maintenance, and expected depreciation add 3,000–5,000 USD per case. In this context, there is a need for more sustainable and cost-effective robotic instrumentation. Published estimates suggest that the SSI Mantra™ has a lower capital cost than some existing platforms. In India, the average reported installation cost for the SSI Mantra™ was approximately 50 million INR (approximately 700,000 USD), which is considerably lower than that of other robotic surgical systems available in India [26]. By offering a platform with potentially lower capital requirements and broad procedural applicability, the system may facilitate wider access to robotic-assisted surgery in resource-constrained settings. However, formal cost-effectiveness analyses are required to fully evaluate its economic impact.

Despite these promising findings, this study had certain limitations. Although the data were prospectively maintained, the retrospective nature of the study limited the ability to infer causal relationships. Surgeon experience was categorized based on cumulative robotic case volumes within this dataset alone; we did not account for broader surgical expertise, prior robotic training on other platforms, or differences in baseline laparoscopic skills. This may have led to the misclassification of experience levels, especially for surgeons with significant prior robotic or minimally invasive surgery experience outside the SSI Mantra™ system. Surgeon self-selection may have skewed outcomes towards high performers, limiting generalizability to less experienced operators. The study included a broad mix of surgical procedures, but some subspecialty and procedure-specific analyses were limited by small case numbers. For lower-volume or overly complex procedures, the observed trends in console time and conversion rates may not be generalizable. Data on BMI, ASA class, prior surgical history, and malignancy status were unavailable for inclusion in adjusted analyses. Hence, the observed associations with surgical specialty and surgeon experience may partly reflect unmeasured differences in patient risk and procedural complexity. While supplementary CUSUM charts were provided for these, they should be interpreted with caution. Formal complication grading (e.g., Clavien-Dindo) was not consistently available across all centers, which limited granular safety comparisons. In the absence of postoperative complication data, a more comprehensive assessment of safety remains limited. Long-term outcomes such as recurrence rates, functional recovery, and patient-reported quality of life were not assessed in this study. This limits conclusions about the sustained effectiveness of the SSI Mantra™ robotic system over time.

## Conclusion

This first-of-its-kind multicenter study from India provides an extensive early evaluation of the SSI Mantra™ robotic system across a large, diverse sample of specialties and surgeons. The platform enabled the feasible completion of simple to complex procedures with minimal conversions and no device-related complications, showing intraoperative safety and feasibility. Its modular arms and unique open-console design support surgeon adaptability and ergonomics, especially in complex multi-quadrant surgeries. Notably, its affordability broadens access to robotic surgery in tier II and tier III cities, helping advance fair surgical care. As adoption grows, future research should assess long-term outcomes, patient satisfaction, low capital cost, and standardized training, including randomized comparisons with conventional laparoscopy, to improve the integration of this indigenous technology.

## Supplementary Information

Below is the link to the electronic supplementary material.

Supplementary Material 1

## Data Availability

Data supporting this study are available from the corresponding author upon reasonable request.
