# Supplementary material for: Real-world performance of the SSI Mantra™ robotic system: a multi-centric multi-specialty study evaluating its safety and surgical applications
Source: J Robot Surg. 2026 Jan 3;20(1):136. doi: 10.1007/s11701-025-03118-2 (PMC12764659; doi:10.1007/s11701-025-03118-2)

**Journal of Robotic Surgery**

**Real-World Performance of the SSI Mantra™ Robotic System: A Multi-Centric Multi-Specialty Study Evaluating its Safety and Surgical Applications**

**Running head:** SSI Mantra™ Robotic System Study

Somashekhar SP^1^, Medha Sugara^1^, Kushal Agrawal^1^, Sudhir Kumar Rawal^2^, Amitabh Singh^2^, Magan Mehrotra^3^, Raj Gajbhiye^4^, Chandramohan Vaddi^5^, Srikarthik Voleti^1^, Leena Mehrotra ^6^, Ganesh Gorthi^7^, Manjiri Somashekhar^8^, Nitin Kumar Rajput^9^

^1^Department of Surgical Oncology, Aster International Institute of Oncology, Bangalore, India.

^2^Department of Genito-Uro-Oncology, Rajiv Gandhi Cancer Institute, New Delhi, India.

^3^Department of Minimal Access and Bariatric Surgery, Apex Hospital, Moradabad, India.

^4^Department of General Surgery, Government Medical College, Nagpur, India.

^5^Department of Urology, Preeti Urology and Kidney Hospital, Hyderabad, India.

^6^Department of Gynecology, Apex Hospital, Moradabad, India.

^7^Department of Surgical Gastroenterology, Continental Hospital, Hyderabad, India.

^8^Department of Pediatric Surgery, Aster International Institute of Oncology, Bangalore, India.

^9^Department of Cardiothoracic Surgery Narayana Institute of Cardiac Sciences, Narayana Health, Bengaluru, India.

**Corresponding author:** Dr. (Prof) Somashekhar SP

Aster International Institute of Oncology, Aster Hospital, Aster CMI Hospital, No. 43/2, New Airport Road, NH44, Sahakar Nagar, Hebbal, Bengaluru, India 560092.

Contact number: +919845712012

Email ID: [drspsomashekhar@gmail.com](mailto:drspsomashekhar@gmail.com)

**Supplementary Table S1**. Distribution of robot-assisted surgeries performed with the SSI Mantra™ system by region and hospital. The table shows the top hospitals by procedure volume in each region (North, South, East, West).

| **Region** | **Sl. No.** | **Hospital** | **Surgery Count** |
| --- | --- | --- | --- |
| **North** | 1 | Rajiv Gandhi Cancer Institute and  Research Center, Delhi NCR | 454 |
|  | 2 | Apex Hospital, Moradabad | 404 |
|  | 3 | Aadhar Health Institute, Hisar | 159 |
|  | 4 | Medanta Hospital, Gurgaon | 59 |
|  | 5 | Meenakshi Hospital, Ghaziabad | 49 |
|  | 6 | Marengo, Gurgaon | 38 |
|  | 7 | Medanta Hospital, Gurgaon | 38 |
|  | 8 | Apex Hospital, Moradabad | 26 |
|  | 9 | Metro Hospital, Faridabad | 26 |
|  | 10 | SRMS, Bareilly | 26 |
| **South** | 1 | Aster-CMI Hospital, Bangalore | 235 |
|  | 2 | Continental Hospital, Hyderabad | 129 |
|  | 3 | Preeti Hospital, Hyderabad | 119 |
|  | 4 | NH, Bangalore | 117 |
|  | 5 | Hindusthan Hospital, Coimbatore | 74 |
|  | 6 | KIIMS Hospital, Hyderabad | 47 |
|  | 7 | Yenepoya Hospital, Mangalore | 31 |
|  | 8 | KLE Hospital, Belgaum | 29 |
|  | 9 | Silverline Hospital | 19 |
|  | 10 | Prakriya Hospital, Bangalore | 17 |
| **East** | 1 | Shanti Memorial Hospital, Cuttack | 53 |
|  | 2 | Ruban Memorial Hospital, Bihar | 30 |
|  | 3 | Healthworld Hospital, Asansol | 16 |
|  | 4 | Fortis Hospital & Kidney Institute, Kolkata | 13 |
|  | 5 | Agile Hospital, Guwahati | 5 |
| **West** | 1 | GMC, Nagpur | 167 |
|  | 2 | Kaizen Hospital, Ahmedabad | 98 |
|  | 3 | Conwest Jain Hospital, Mumbai | 76 |
|  | 4 | Sanjeevani Hospital, Raipur | 63 |
|  | 5 | Cytocure Hospital, Mumbai | 61 |
|  | 6 | Marengo, Ahmedabad | 29 |
|  | 7 | Dhamelia Hospital, Surat | 26 |
|  | 8 | ICON Hospital, Surat | 25 |
|  | 9 | GBL Hospital, Indore | 23 |
|  | 10 | Noble Hospital, Pune | 18 |

**Supplementary Table S2**. Experience and conversion-wise length of stay.

| **Experience Group** | **Conversion Status** | **n** | **% of Group** | **Median LOS (IQR)** | **Mean LOS (SD)** | **Min–Max** |
| --- | --- | --- | --- | --- | --- | --- |
| Q1–Q3 | Not Converted | 485 | 92.9% | 3 (2–4) | 2.99 (2.04) | 1–38 |
| Q1–Q3 | Converted | 37 | 7.1% | 3 (2–4) | 5.00 (9.48) | 1–60 |
| Q4 | Not Converted | 1997 | 96.3% | 3 (2–4) | 2.96 (1.40) | 1–16 |
| Q4 | Converted | 77 | 3.7% | 3 (2–3) | 3.29 (2.22) | 1–15 |

Q: quartile; IQR: interquartile range; SD: standard deviation; LOS: length of stay; Min: minimum; Max: maximum

**Supplementary Table S3**. Multivariable linear regression of console time (minutes).

| **Variable** | **β (minutes)** | **95% Confidence Interval** | **p-value** |
| --- | --- | --- | --- |
| Conversion to open surgery (Yes vs No) | −36.7 | −52.1 to −21.3 | <0.001 |
| Surgeon experience (More vs Less experienced) | −28.4 | −35.8 to −20.9 | <0.001 |
| Cardiac | −73.5 | −161.0 to 13.8 | 0.099 |
| Colorectal | 11.0 | −76.4 to 98.4 | 0.806 |
| Gastrointestinal | 8.5 | −79.7 to 96.7 | 0.850 |
| General | −98.7 | −185.0 to −12.2 | 0.025 |
| Gynecology | −46.0 | −133.0 to 40.7 | 0.298 |
| Head and neck | −49.1 | −139.0 to 40.9 | 0.285 |
| Thoracic | −60.6 | −153.0 to 32.2 | 0.200 |
| Urology | 3.4 | −83.2 to 89.9 | 0.939 |

**Supplementary Table S4**. Log-transformed multivariable regression of console time (Sensitivity analysis; relative effects).

| **Variable** | **β (log scale)** | **95% Confidence Interval** | **p-value** | **Approx. % Change** |
| --- | --- | --- | --- | --- |
| Conversion to open surgery (Yes vs No) | −0.235 | −0.353 to −0.117 | <0.001 | −21% |
| Surgeon experience (More vs Less experienced) | −0.364 | −0.421 to −0.306 | <0.001 | −30% |
| Cardiac | −0.705 | −1.37 to −0.038 | 0.038 | −50% |
| Colorectal | −0.048 | −0.715 to 0.619 | 0.887 | −5% |
| Gastrointestinal surgery | −0.222 | −0.895 to 0.451 | 0.518 | −20% |
| General surgery | −0.967 | −1.63 to −0.307 | 0.004 | −62% |
| Gynecology | −0.409 | −1.07 to 0.253 | 0.226 | −34% |
| Head and neck | −0.451 | −1.14 to 0.236 | 0.198 | −36% |
| Thoracic | −0.450 | −1.16 to 0.258 | 0.213 | −36% |
| Urology | −0.065 | −0.726 to 0.595 | 0.846 | −6% |

**Supplementary Figure S1**. Residual plots to inspect deviations from linearity or homoscedasticity

**
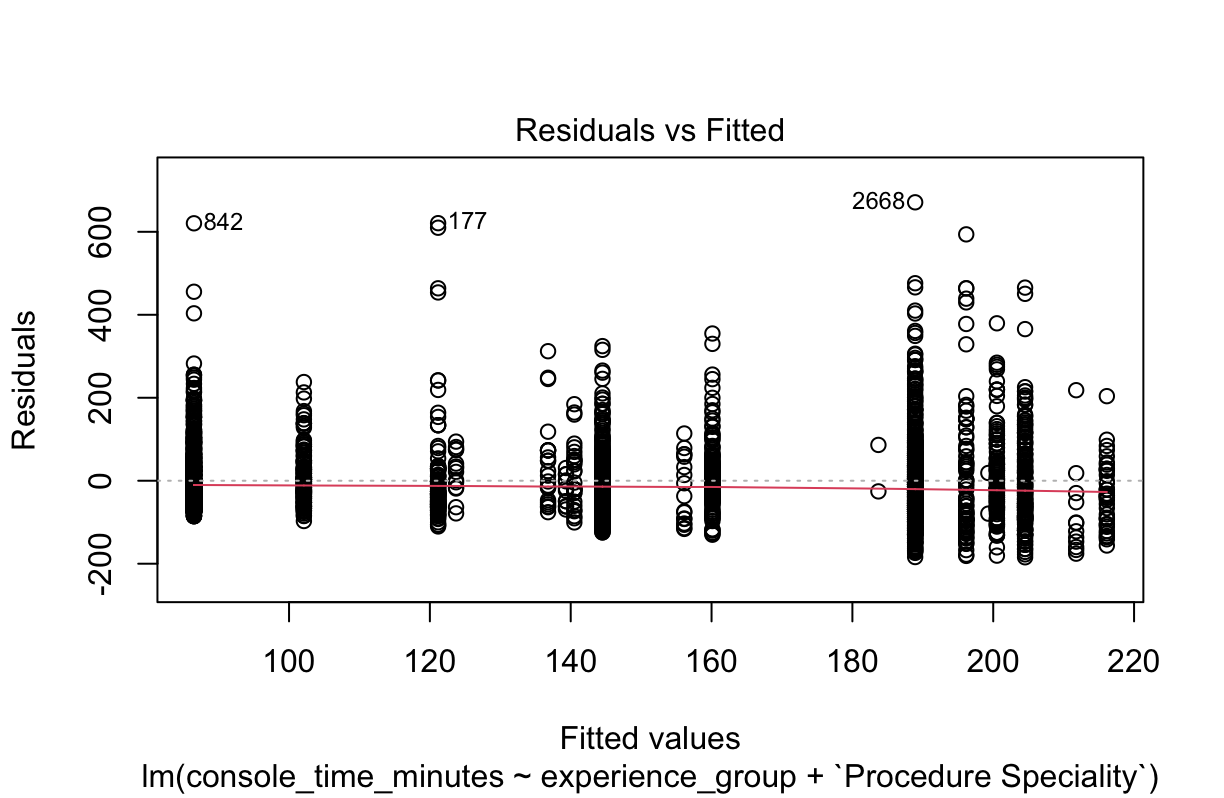
**

**Supplementary Figure S2**. Conversion rate (%) by surgical procedure with ≥20 cases and ≥5 conversions

**
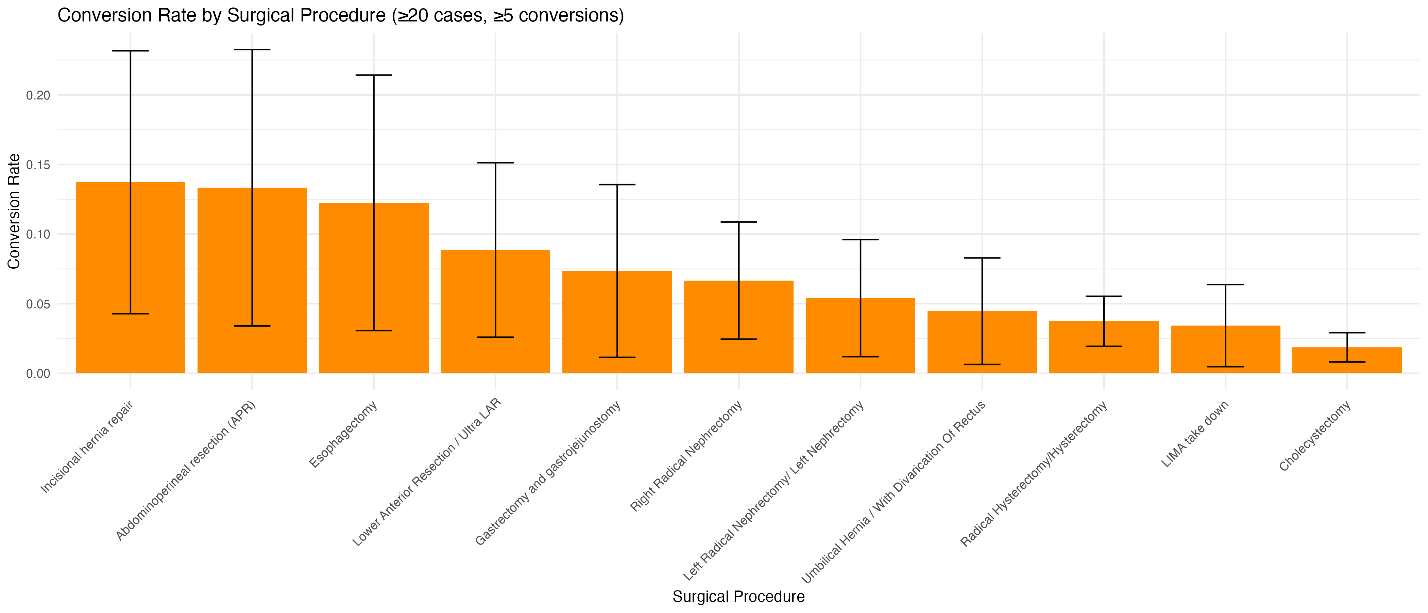
**

**Supplementary Figure S3**. Continuous CUSUM charts for lower-volume procedures. **a** Internal mammary artery (IMA) harvest (cardiac), **b** Lower anterior resection (colorectal), **c** Hysterectomy (gynecology), **d** Gastrectomy/gastrojejunostomy (gastrointestinal)


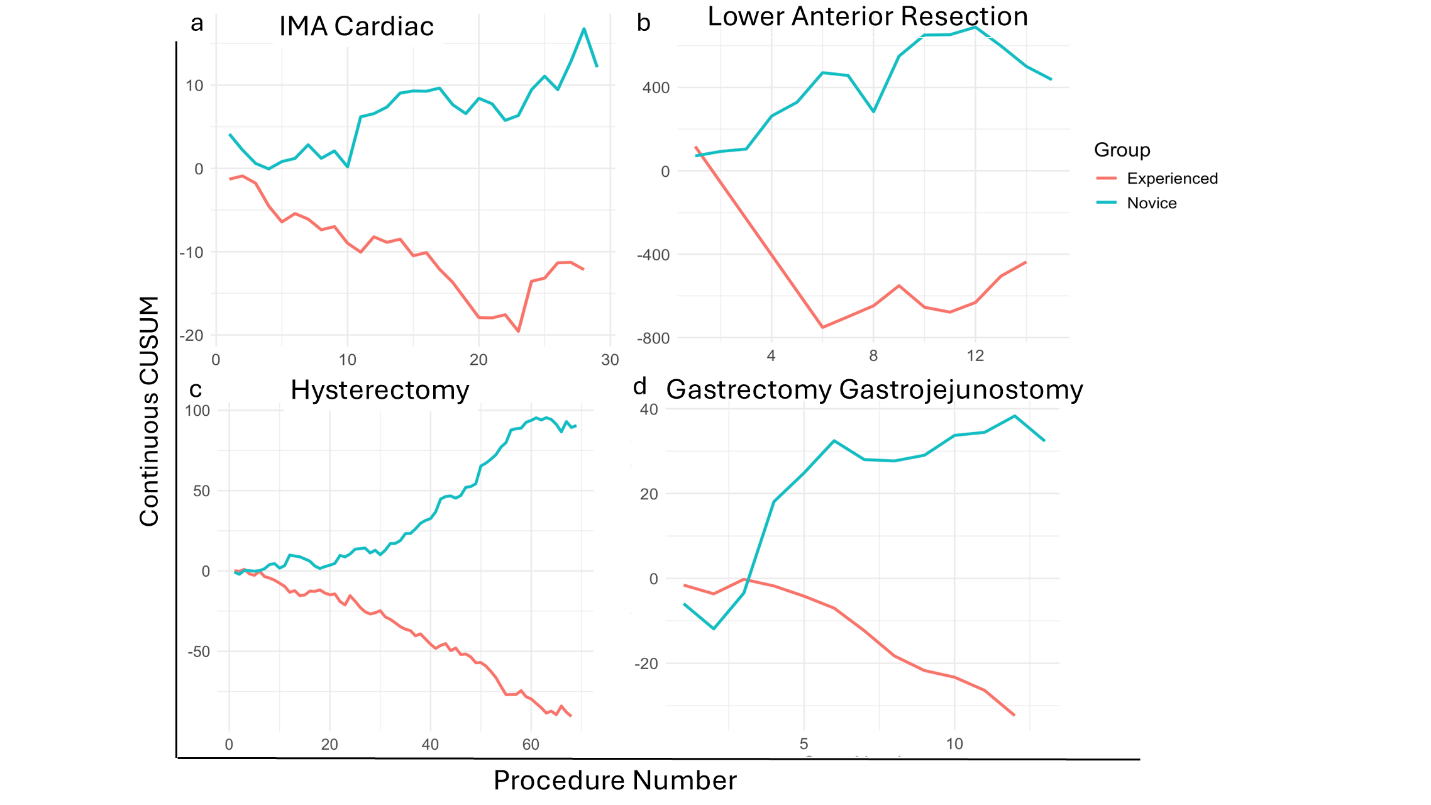


**Supplementary Figure S4**. **a** & **b** Radar plots comparing surgeon experience groups on raw and normalized values for console time, conversion rate, and length of stay, respectively


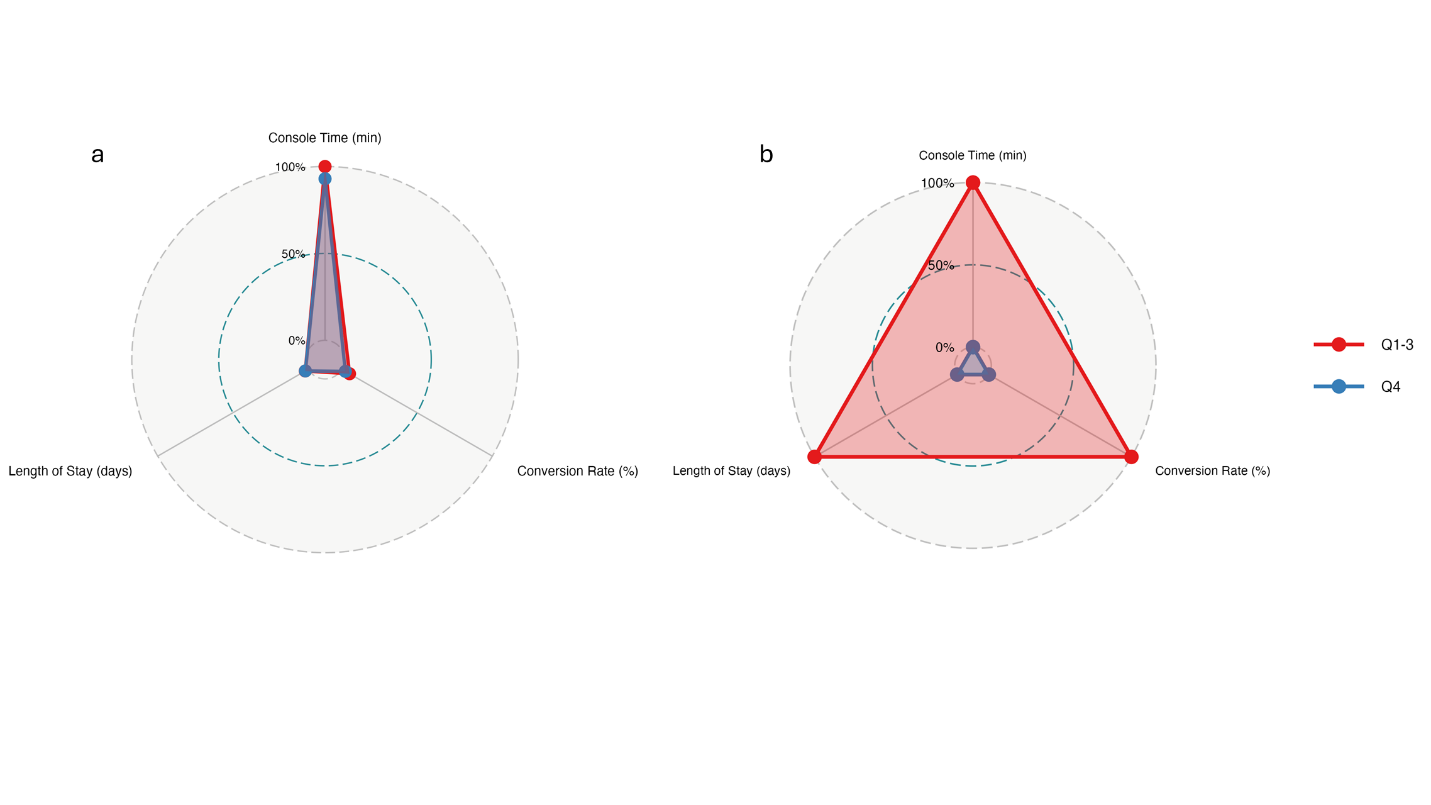

Supplement: Supplementary file 1 — Supplementary Material 1 [file 11701_2025_3118_MOESM1_ESM.docx]
